# Supplementary material for: Technical Complications of Removable Partial Dentures in the Moderately Reduced Dentition: A Systematic Review
Source: Dent J (Basel). 2023 Feb 20;11(2):55. doi: 10.3390/dj11020055 (PMC9955676; doi:10.3390/dj11020055)
Supplement: Supplementary file 1 [file dentistry-11-00055-s001.zip › dentistry-2199174-supplementary.pdf]

dentistry journal

Supplementary Materials: The following supporting information can be downloaded at: www.mdpi.com/xxx/s1

Table S1-1. Used search terms

Table S1-2. Used electronic databases

Table S1-3. Sources for the hand search until January, 15th 2014

Table S2-1. Risk of bias for RCTS with intention-to-treat based on the Cochrane library

Table S2-2. Risk of bias for RCTS with pre-protocol based on the Cochrane library

Table S3. Risk of bias for non-randomized studies of interventions based on the Cochrane library

Table S4-1. Summary of the results of the included studies: clasp-retained RPDs

Table S4-2. Summary of the results of the included studies: attachment-retained RPDs

Table S4-3. Summary of the results of the included studies: conical double-crown-retained RPDs

Table S4-4. Summary of the results of the included studies: electroplated double-crown-retained RPDs

Table S4-5. Summary of the results of the included studies: double-crown-retained RPDs

Table S4-6. Summary of the results of the included studies: other RPDs (bars, etc.)

Table S1. Used search terms .

Number	Used search terms
1	Denture, Partial, Removable [MeSH Terms] AND (Short* dent* arch*)
2	Denture, Partial, Fixed [MeSH Terms] AND (Short* dent* arch*)
3	Denture, Partial, Fixed [MeSH Terms] AND (clinical AND (trial OR study))
4	Denture, Partial, Removable [MeSH Terms] AND (clinical AND (trial OR study))
5	Denture, Partial, Fixed [MeSH Terms] AND (cantil* OR extens*)
6	Denture, Partial, Fixed [MeSH Terms] AND "Dental Prosthesis, Implant-Supported" [Mesh] AND (teeth OR tooth OR molar OR premolar) AND (missing OR posterior)
7	Denture, Partial, Fixed [MeSH Terms] AND "Dental Prosthesis, Implant-Supported" Mesh] AND (clinical AND (trial OR study))
8	Denture, Partial, Fixed [MeSH Terms] AND Dental Implants [MeSH Terms] AND (clinical AND (trial OR study))
9	Denture, Partial, Fixed [MeSH Terms] AND Dental Implants [MeSH Terms] AND (teeth OR tooth OR molar OR premolar) AND (missing OR posterior)
10	"Denture, Partial, Removable" [MeSH Terms] AND "Dental Prosthesis, Implant-Supported" [MeSH Terms]
11	"Denture, Partial, Removable" [MeSH Terms] AND "Dental Implants"[Mesh]
12	((teeth OR tooth OR molar OR premolar) AND (missing OR posterior)) AND (clinical AND (trial OR study))
13	((teeth OR tooth OR molar OR premolar) AND (missing OR posterior)) AND ((Denture, Partial, Fixed [MeSH Terms]) OR (Denture, Partial, Removable [MeSH Terms]) OR (Dental Prosthesis, Implant-Supported [MeSH Terms]) OR (Dental Implants [MeSH Terms]) OR (Short* dent* arch*) OR (cantil* OR extens*)) AND (clinical AND (trial OR study))
14	((Removable AND partial AND denture) AND (trial OR study))

Table S1. Used electronic databases **.**

Number	Used electronic databases
**1**	MEDLINE/PubMed
**2**	EMBASE/Ovid
**3**	BIOSIS/Ovid
**4**	SciSearch/DIMDI
**5**	Cochrane/Cochrane
**6**	FIZ Technik Web/FIZ Technik

Table S1. Sources for the hand search until January, 15th 2014.

Number	Sources for the hand search until January, 15th 2014
**1**	Clinical Oral Investigations, 1997–date http://link. springer.com/journal/volumesAndIssues/784
**2**	The International Journal of Prosthodontics, 1988–date http://www.quintpub.com/journals/ijp/gp.php?journal_ name=ijp
**3**	The Journal of Prosthetic Dentistry, 1950–date http:// www.journals.elsevierhealth.com/periodicals/ympr/issues
**4**	Deutsche Zahnärztliche Zeitschrift, 1945–date (also avail- able online from 2001 onwards) www.zahnheilkunde.de &
**5**	Schweizer Monatsschrift für Zahnmedizin (Swiss Dental Journal), 1987–date (also available online from 1997 on- ward) www.sso.ch
**6**	Journal of Dentistry, 1972–date www.jodjournal.com/ issues
**7**	Journal of Dental Research, 1919–date http://jdr.sagepub. com/content/by/year

Table S2. Risk of bias for RCTS with intention-to-treat based on the Cochrane library (based on Sterne JAC, Savović J, Page MJ, Elbers RG, Blencowe NS, Boutron I, Cates CJ, Cheng H-Y, Corbett MS, Eldridge SM, Hernán MA, Hopewell S, Hróbjartsson A, Junqueira DR, Jüni P, Kirkham JJ, Lasserson T, Li T, McAleenan A, Reeves BC, Shepperd S, Shrier I, Stewart LA, Tilling K, White IR, Whiting PF, Higgins JPT. RoB 2: a revised tool for assessing risk of bias in randomised trials. *BMJ* 2019; **366**: l4898).

Author, year	Weight	D1	D2	D3	D4	D5	Overall			
Budtz-Jorgensen, 1987	1	!	!	+	+	!	!		+	Low risk
Budtz-Jorgensen, 1990	1	!	!	+	+	!	!		!	Some concerns
Stober, 2012	1	!	!	+	+	!	!			High risk
Stober, 2015	1	!	!	+	+	!	!			
Wolfart, 2012	1	+	!	+	+	!	!		D1	Randomisation process
									D2	Deviations from the intended interventions
									D3	Missing outcome data
									D4	Measurement of the outcome
									D5	Selection of the reported result

Table S2. Risk of bias for RCTS with pre-protocol based on the Cochrane library (based on Sterne JAC, Savović J, Page MJ, Elbers RG, Blencowe NS, Boutron I, Cates CJ, Cheng H-Y, Corbett MS, Eldridge SM, Hernán MA, Hopewell S, Hróbjartsson A, Junqueira DR, Jüni P, Kirkham JJ, Lasserson T, Li T, McAleenan A, Reeves BC, Shepperd S, Shrier I, Stewart LA, Tilling K, White IR, Whiting PF, Higgins JPT. RoB 2: a revised tool for assessing risk of bias in randomised trials. *BMJ* 2019; **366**: l4898).

**Per-protocol**	**Author, year**	**Weight**	**D1**	**D2**	**D3**	**D4**	**D5**	**Overall**			
	**Au, 2000**	**1**	!	!	+	+	!	!		+	**Low risk**
	**Kapur, 1989, Part II**	**1**	!	+	+	+	!	!		!	**Some concerns**
	**Kapur, 1994, Part I**	**1**	!	+	+	+	!	!			**High risk**
	**Schwindling, 2017**	**1**	+	+	+	!	!	!			
	**Thomason, 2007**	**1**	!	!	+	+	!	!		**D1**	**Randomisation process**
										**D2**	**Deviations from the intended interventions**
										**D3**	**Missing outcome data**
										**D4**	**Measurement of the outcome**
										**D5**	**Selection of the reported result**

Table S3. Risk of bias for non-randomized studies of interventions based on the Cochrane libraray (based on Sterne JAC, Hernán MA, Reeves BC, Savović J, Berkman ND, Viswanathan M, Henry D, Altman DG, Ansari MT, Boutron I, Carpenter JR, Chan AW, Churchill R, Deeks JJ, Hróbjartsson A, Kirkham J, Jüni P, Loke YK, Pigott TD, Ramsay CR, Regidor D, Rothstein HR, Sandhu L, Santaguida PL, Schünemann HJ, Shea B, Shrier I, Tugwell P, Turner L, Valentine JC, Waddington H, Waters E, Wells GA, Whiting PF, Higgins JPT. ROBINS-I: a tool for assessing risk of bias in non-randomized studies of interventions. BMJ 2016; 355; i4919; doi: 10.1136/bmj.i4919.).

Author, year	Bias due to confounding	Bias in selection of participants into the study	Bias in classification of interventions	Bias due to deviations from intended interventions	Bias due to missing data	Bias in measurement of outcomes	Bias in selection of the reported result	Overall bias
Al-Imam, 2016	Moderate	Low	Low	Low	Moderate	Low	Low	Moderate
Behr, 2000	Moderate	Moderate	Low	Low	Low	Low	Low	Moderate
Behr, 2009	Moderate	Moderate	Low	Low	Low	Low	Low	Moderate
Behr, 2012	Moderate	Moderate	Low	Low	Low	Low	Low	Moderate
Bergman, 1971	Moderate	Low	Low	Low	Low	Low	Low	Moderate
Bergman, 1977	Moderate	Low	Low	Low	Moderate	Low	Low	Moderate
Bergman, 1982	Moderate	Low	Low	Low	Low	Low	Low	Moderate
Bergmann, 1996	Moderate	Low	Low	Low	Moderate	Low	Low	Moderate
Ericson, 1990	Moderate	Low	Low	Low	Moderate	Low	Low	Moderate

Hahnel,2012	Moderate	Low	Low	Low	Moderate	Low	Low	Moderate
Heydecke, 2003	Moderate	Moderate	Low	Low	Moderate	Low	Low	Moderate
Ishida, 2016	Moderate	Low	Low	Low	Moderate	Low	Low	Moderate
Kurosaki, 2021	Moderate	Moderate	Low	Low	Moderate	Low	Low	Moderate
Mock, 2005	Moderate	Low	Low	Low	Low	Low	Low	Moderate
Nickenig, 1995	Moderate	Moderate	Low	Low	Low	Low	Low	Moderate
Nisser, 2022	Moderate	Moderate	Low	Low	Low	Low	Low	Moderate
Pihlaja, 2015	Moderate	Moderate	Low	Low	Low	Low	Low	Moderate
Rehmann, 2006	Moderate	Moderate	Low	Low	Low	Low	Low	Moderate
Schmitt, 2011	Moderate	Low	Low	Low	Low	Low	Low	Moderate
Scholz, 2010	Moderate	Low	Low	Low	Low	Low	Low	Moderate

Schulte, 1980	Moderate	Moderate	Low	Low	Moderate	Low	Low	Moderate
Schwindling, 2014	Moderate	Moderate	Low	Low	Moderate	Low	Low	Moderate
Stegelmann,2012	Moderate	Moderate	Low	Low	Low	Low	Low	Moderate
Stober, 2020	Moderate	Low	Low	Low	Low	Low	Low	Moderate
Vanzeveren, Part I,2003	Moderate	Moderate	Low	Low	Moderate	Low	Low	Moderate
Vanzeveren, Part II,2003	Moderate	Moderate	Low	Low	Moderate	Low	Low	Moderate
Vermeulen, 1996	Moderate	Moderate	Low	Low	Moderate	Low	Low	Moderate
Wagner, 2000	Moderate	Moderate	Low	Low	Moderate	Low	Low	Moderate
Wenz, 2001	Moderate	Moderate	Low	Low	Low	Low	Low	Moderate

Widbom, 2004	Moderate	Moderate	Low	Low	Moderate	Low	Low	Moderate
Wöstmann, 2007	Moderate	Moderate	Low	Low	Moderate	Low	Low	Moderate
Yoshino, 2019	Moderate	Moderate	Low	Low	Low	Low	Low	Moderate
Zierden, 2018	Moderate	Moderate	Low	Low	Moderate	Low	Low	Moderate

Table S4. Summary of the results of the included studies: clasp-retained RPDs.

Author, year	Retention of prosthesis	Retention loss of anchor crowns (decementation)	Fracture of the framework (including the anchorage elements and their repair)	Fracture/repair of denture teeth or veneering	Fracture/repair of acrylic denture base/saddles	Relining/rebasing	Other failures/complications
Al-Imam, 2016 **[5]**			Fracture of clasps: 3 (4.6%) of patients		Fracture of acrylic: 1 (1.5%) of patients		Ill-fitting protheses in 16 (24.6%) patients (lack of differentiation)
Au, 2000 **[6]**	Inadequate retention: 3 (10.7%) of cobalt-chromium RPDs		Fracture of occlusal rest: 1 (5.3%) of Titanium-RPDs; 1 (3.6%) of Cobalt-chromium RPDs. Fracture of minor connector: 1 (5.3%) of Titanium-RPDs	Denture tooth debonding: 1 (5.3%) of Titanium-RPDs, 1 (3.6%) of Cobalt-chromium RPDs	Porosity of acrylic: 1 (5.3%) of Titanium-RPD, 1 (3.6%) of Cobalt-chromium RPDs		Retainer not connecting to tooth: 2 (10.5%) of Titanium-RPDs, 6 (21.4%) of Cobalt-chromium-RPDs. Poor clinical fit: 1 (5.3%) of Titanium-RPDs. High occlusion: 1 (3.6%) of Cobalt-chromium-RPDs. Out of occlusion: 1 (3.6%) of Cobalt-chromium-RPDs
Behr, 2012 **[9]**			Clasps fracture: 28 (16.1%) of RPDs. Fracture of major connector: 9 (5.1%) of RPDs. Fracture of minor connector: 6 (3.4%) of RPDs	Loss of artificial acrylic resin teeth: 8 (4.6%) of RPDs. Considerable wear of acrylic resin		Relining necessary: 32 (18.3%) of RPDs	

				teeth: 6 (3.4%) of RPDs			
Bergmann, 1971 **[10]**	Impaired retention: 6 (20.7%) of patients		Fracture of lingual bar: 1 (3.5%) of patients. Clasp fracture: 1 (3.5%) of patients	Loss of tooth in the denture: 1 (3.5%) of patients	Crack in the base of one denture: 1 (3.5%) of patients		
Bergmann, 1977 **[11]**	Impaired retention: 2-4 years: 10 (35.7%) of patients; 4-6 years: 11 (39.3%) of patients		Clasp adjustment: 32 (no percentage can be given because only the number of patients followed up (n=28) is given, but not the number of prostheses followed up or the number of follow-up treatments per patient or prosthesis). Clasp replacement: 5. New lower partial dentures due to fracture of the casting: 1. Minor repairs of the partial denture casting: 2	Renewals of porcelain or acrylic facings due to fracture or abrasion: 3. Dentures requiring replacement of teeth due to abrasion: 3	Denture base adjustments: 16	Rebasings or relinings and carried out in conjunction with occlusal grinding: 19	
Bergmann, 1982 **[12]**			Remade because of framework fracture: 2 (no percentage can be given because only the number of patients followed up (n=27) is given, but not the number of prostheses	Exchange of facings: 5	Minor repairs of the acrylic part of the denture: 44	Rebasing, including grinding (occlusal correction): 28	

			followed up or the number of follow-up treatments per patient or prosthesis). Remade because of loss of framework fit: 7. Remade because of instability caused by poor fit of the framework and denture base: 3. Repair of framework: 2				
Budtz-Jørgensen, 1987 **[14]**			Repair of framework: 2 (no percentage can be given, as only the number of findings/measures is given and not a number of patients or prosthesis affected). Adjustment of sublingual bar: 10. Fracture of major connector: 2. Clasp fracture: 2		Minor repairs of the acrylic part of the dentures: 8	Relining: 5	Occlusal grinding: 5
Budtz-Jørgensen, 1990 **[15]**			Clasp fractures: 4 ((no percentage can be given, as only the number of findings/measures is given and not a number of patients or prostheses affected). Major repair of framework: 6	Denture failures: 10		Relining: 7	
Ishida, 2017 **[19]**		Loss of cementation:	Complications of the retainer: 12 (6.0%) of	Complications of	Complications of		

	15 (3.8%) of the abutment teeth. This corresponded to 15 (28.3%) of 53 complications of abutment teeth	RPDs. This corresponded to 12 (11.1%) of 108 complications of RPDs	artificial tooth: 15 (7.5%) of RPDs. This corresponded to 15 (13.9%) of 108 complications of RPDs	dentures base: 67 (33.7%) of RPDs. This corresponded to 67 (62%) of 108 complications of RPDs	
Kapur, 1989 **[20]**		Broken or distorted frameworks or lost dentures: 11 (9.3%) of RPDs			Rebasing: 23 (19.5%) der RPDs
Kapur, 1994 **[21]**		Remake because of broken metal framework: 1 (1.7%) of the RPDs with circumferential design. Remake because of bent: 3 (5.1%) of RPDs with circumferential design; 3 (5.1%) of RPDs with bar design			Rebasing: 15 (25.4%) of RPDs w/ circum-ferential design; 8 (13.6%) of RPDs w/ bar design. Rebased and/or remade: 7 (11.9%) of RPDs w/ circumferential design; 7 (11.9%) of RPDs w/ bar design
Kurosaki, 2021 **[22]**			Fracture of the superstructure	Loss of prothesis because of	

				veneering material: 0 (0%) of patients	extraction of abutment teeth, fracture of the denture base, discomfort from using RPD: 15 (75%) of patients		
Nisser, 2022 **[25]**			Fracture of the framework: 3 (1.7%) of RPDs. Fracture of clasps leading to failure: 9 (5.2%) of the RPDs. Fracture of clasps not leading to failure: 8 (4.7%) of RPDs. Combined complications: Fracture of claps and fracture of acrylic teeth: 2 (1.2%) of RPDs; fracture of clasps and loss of natural teeth: 1 (0.6%) of RPDs	Loss/fracture of prosthetic acrylic teeth: 5 (2.9%) of RPDs. Combined complications: loss of natural teeth and fracture of acrylic teeth: 2 (1.2%) of RPDs		Need for relining of the prothesis base: 4 (2.3%) of RPDs	Failure because of poor fit and adaptation: 24 (14.0%) of RPDs. Need for addition of more prosthetic elements, leading to failure: 1 (0.6%) of RPDs
Pihlaja, 2015 **[26]**	Good retention (= resisted replacement): 17 (100%) of RPDs		Crown of abutment teeth with fracture of occlusal rest seat: 1 (2.7%) of abutment teeth	Fracture of the veneering porcelain: 2 (11.8%) of RPDs			
Stegelmann, 2012 **[33]**			Fracture of metal framework: 5 (7.4%) of RPDs	Repair of facing: 3 (4.4%) of RPDs	Fracture of the denture base: 2 (2.9%) of the		

			RPDs. Cracks in denture base: 1 (1.5%) of RPDs	
Thomason, 2007 **[37]**	Distortion, removable partial denture fracture, loss of tooth: 10 (5.7%) of the 175 treatment needs. Removable partial denture adjustments: 60 (34.3%) of the treatment needs			
Vanzeveren, Part I, 2003 **[38]**	Fracture of the metal saddle: 7 (2.4%) of RPDs	Repair of saddles and artificial teeth: 7 (2.4%) of RPDs. Refixation of a tooth: 18 (6.2%) of RPDs. Replacement of a tooth: 4 (1.4%) of RPDs. Replacement of the saddle and the teeth: 5 (1.7%) of RPDs		Relines materialized: 30 (10.3%) of RPDs. Relines recommended: 32 (11%) of RPDs

Vanzeveren, Part II, 2003 **[39]**		Fractures of the retentive systems: 27 (3.4%) of the abutment teeth		
Vermeulen, 1996 **[40]**		Survival related to fracture of prosthetic framework: extension base: upper after 5 years: 84 ± 5%; upper after 10 years: 65 ± 9%; lower after 5 years: 86 ± 2%; lower after 10 years: 72 ± 4%; tooth-supported base: upper after 5 years: 89 ± 3%; upper after 10 years: 73 ± 5%; upper after 5 years: 82 ±4 %; upper after 10 years: 56 ±7 %	Survival related to adjustment denture base: extension base: upper after 5 years: 60 ± 6%; upper after 10 years: 40 ± 9%; lower after 5 years: 65 ± 3%; lower after 10 years: 41 ± 4%; tooth-supported base: upper after 5 years: 82 ± 3%; upper after 10 years: 55 ± 6%; Lower after 5 years: 75 ± 4 %; lower after 10 years: 55 ± 7 %	

Wagner, 2000 **[41]**	Retention: 1-5 N (mean 2.3 N)

Table S4. Summary of the results of the included studies: attachment-retained RPDs.

Author, year	Retention of prosthesis	Retention loss of anchor crowns (decementation)	Fracture of the framework (including the anchorage elements and their repair)	Fracture/repair of denture teeth or veneering	Fracture/repair of acrylic denture base/saddles	Relining/ rebasing	Other failures/ complications
Heydecke, 2003 **[18]**		Decementation: after in mean 27 months: 4 (7.3%) of RPDS; after 59 ± 11 months: 7 (12.7%) of the RPDs (both percentages refer to the number (n=55) of prostheses reexamined after an average of 27 months)	Fracture of the metal: after in mean 27 months: 2 (3.6%) of RPDs; after 59 ± 11 months: 2 (3.6%) of RPDs. Need to replace a retention element (15 due to spring fractures): after in mean 27 months: 14 (25.5%) of RPDS; after 59 ± 11 months: 20 (36.4%) of RPDs. Fractures or corrosion of the attachment: after in mean 27 months: 3 (5,5%) of RPDs; after 59 ± 11 months: 6 (10,9%) of RPDs	Chipped denture teeth: after in mean 27 months: 4 (7.3%) of RPDs; after 59 ± 11 months: 8 (14.5%) of RPDs. Chipped facings: after in mean 27 months: 5 (9.1%) of the RPDs; after 59 ± 11 months: 5 (9.1%) of RPDs	Fracture of the denture base: after in mean 27 months: 4 (7.3%) of RPDs; after 59 ± 11 months: 6 (10.9%) of the RPDs	Relining because of bone resorption: after in mean 27 months: 10 (18.2%) of the RPDS; after 59 ± 11 months: 10 (18.2%) of RPDs	
Schmitt 2011 **[28]**	Attachment screw activation: RDPs	Decementation: RPDs with		Fracture of the ceramic			

	with extracoronal attachment with interchangable plastic inserts that are adjustable with activation screw: 20 (46.5%) of attachments; RDPs with spring bolt attachments: 3 (37.5%) of attachments. Change of plastic insert of RPD with extracoronal attachment with interchangable plastic inserts that are adjustable with activation screw: 17 (39.5%) of attachments. Irreversible mechanical wear of attachment of the male part of the spring bolt attachment: 4 (50%) of attachments	extracoronal attachment with interchangable plastic inserts that are adjustable with activation screw): 1 (2.3%) of attachments; RPDs with spring bolt attachments: 1 (12.5%) of attachments		veneering: RPDs with spring bolt attachments: 3 (37.5%) of attachments		
Stegelmann, 2012 [33]			Fracture of metal framework: 3 (2.2%) of RPDs	Repair of a facing: 5 (3.7%) of RPDs	Fracture of the denture base: 5 (3.7%) of RPDs. Crack in the denture base: 1 (0.7%) of RPDs	

Wolfart, 2012 [44]	Change of plastic insert: 32 (39.5%) of RPDs, which corresponded to 34 treatments (7.4% of all 459 treatments)	Decementation: 13 (16%) of the RPDs, which corresponded to 19 treatments (4.1% of all treatments)	Fracture of framework: 0 (0%) of the RDPs. Repair of frameworks or minor connector: 5 (6.2%), which corresponded to treatments (1.3% of all treatments)	Facing renewed: 12 (14.8%) of RPDs, which corresponded to 15 treatments (3.3% of all treatments). Renewal of acrylic teeth: 4 (4.9%) of RPDs, which corresponded to 5 treatments (1.1% of all treatments)	Repair of denture base: 1 (1.2%) of the RPDs, which corresponded to 2 treatments (0.4% of all treatments)	Relining: 27 (33.3%) of RPDs, which corresponded to 29 treatments (6.3% of all treatments)	Occlusal adjustments: 7 (8.6%) of the RPDs, which corresponded to 12 treatments (2.6% of all treatments)

Table S4. Summary of the results of the included studies: conical double-crown-retained RPDs. 26

Author, year	Retention of prothesis	Retention loss of anchor crowns (decementation)	Fracture of the framework (including the anchorage elements and their repair)	Fracture/repair of denture teeth or veneering	Fracture/repair of acrylic denture base/saddles	Relining/rebasing	Other failures/ complications
Behr, 2000 [7]		Decementation: 8 (18.6%) of RPDs	Fracture of the metal framework: 3 (7%) of RPDs	Loss of facings: 4 (9,3%) of RPDs. Fracture of artificial teeth: 3 (7%) of the RPDs	Fracture of resin framework: 2 (4.7%) of RPDs		Fracture of soldering: 1 (2,3%) of RPDs
Behr, 2009 [8]		Decementation: 33 (53.2%) of RPDs					
Bergmann, 1996 [13]	Marked retention; extremely marked retention (grades 2-3): 13 (72%) of RPDs	Decementation: 8 (44.4%) of patients, 13 (16.7%) of the abutment teeth			Acrylic resin denture repairs: 17 (no percentage can be given because the authors did not specify how many prostheses or patients this number refers to)		
Ericson, 1990 [16]	Marked retention; extremely marked retention (grades 2-3):			Cervical cracks in the veneering material: 23 (24%) of the secondary crowns			

	21 (87.5%) of RPDs		
Hahnel, 2012 [17]			Failure of veneering: 17 (27.9%) of RPDs
Scholz, 2010 [29]			Estimated fracture rate after 5 years: 20.6% (14.2-26.9%) (percentages refer to Kaplan-Meier estimators with 95% confidence intervals); anterior teeth 17.9% (11.8-24%); premolars 26.4% (7.7-45.1%); molars 8.3% (0-22.3%). Estimated discoloration rate after 5 years: 16.3% (11.2-21.4%)
Stober, 2012 [34]		Decementation: 7 (6.3%) of the primary crowns	Loss/fracture of facings: 10 (8.9%) of the primary crowns
Stober, 2015 [35]		Decementation: 10 (8.9%) of the primary crowns	Loss/fracture of facings:30 (26.8%) of the primary crowns
Wagner, 2000 [41]	Retention: 1-10 N (mean 4.9 N)		

Table S4. Summary of the results of the included studies: electroplated double-crown-retained RPDs.

Author, year	Retention of prothesis	Retention loss of anchor crowns (decementation)	Fracture of the framework (including the anchorage elements and their repair)	Fracture/repair of denture teeth or veneering	Fracture/repair of acrylic denture base/saddles	Relining/rebasing	Other failures/ complications
Schwindling, 2017 [32]		Decementation: 4 (14.3%) of RPDs with Cobalt-chromium primary crowns		Veneer failure: 5 (18.2%) of RPDs with zirconia primary crowns; 5 (17.9%) of RPDs with Cobalt-chromium primary crowns. Combined complications: veneer failure and fracture of a primary crown: 1 (3.7%) of RPDs with zirconia primary crowns; veneer failure and need for relining: 1 (3.6%) of RPDs with Cobalt-chromium primary crowns. Veneer fracture and denture base fracture: 1 (3.6%) of RPDs with Cobalt-chromium primary crowns	Denture base fracture: 3 (11.1%) of RPDs with zirconia primary crowns; 2 (7.1%) of RPDs with Cobalt-chromium primary crowns	Need for relining: 1 (3.7%) of RPDs with zirconia primary crowns; 2 (7.1%) of RPDs with Cobalt-chromium primary crowns	Failure of electroplated structure: 1 (3.6%) of RPDs with Cobalt-chromium primary crowns
Stober, 2012 [34]	Remade because of loss of retention: 1 (3.3%) of RPDs	Decementation: 6 (5.7%) of primary crowns		Losses or fractures of facings: 16 (15.2%) of primary crowns			

Stober, 2015 [35]	Remade because of combination of loss of retention and loss of facings: 1 (3.3%) of RPDs	Decementation: 10 (9.5%) of primary crowns	Losses or fractures of facings: 33 (31.4%) of primary crowns

Table S4. Summary of the results of the included studies: double-crown-retained RPDs.

Author, year (specifics anchoring form)	Retention of prothesis	Retention loss of anchor crowns (decementation)	Fracture of the framework (including the anchorage elements and their repair)	Fracture/repair of denture teeth or veneering	Fracture/repair of acrylic denture base/saddles	Relining/ rebasing	Other failures/ complications
Behr, 2000 (parallel-sided double crowns) [7]		Decementation: 19 (26%) of RPDs	Fracture of metal framework: 2 (2.7%) of RPDs	Loss of facings: 0 (0%) of RPDs. Fracture of artificial teeth: 4 (5.5%) of RPDs			Fracture of soldering: 0 (0%) of RPDs
Behr, 2009 [8]		Decementation: 67 (21.3%) of parallel-sided telescopic crowns with clearance fit; 64 (32%) of parallel-sided telescopic crowns with friction fit					
Hahnel, 2012 [17]				Failure of veneering: parallel-sided telescopic with clearance fit: 14 (4.4%) of RPDs; parallel-sided			

				telescopic with friction fit: 14 (7%) of RPDs	
Ishida, 2017 [19]		Decementation: 20 (13.8%) of abutment teeth. This corresponded to 20 (76.9%) of 26 complications of abutment teeth	Complications of retainer: 3 (5.8%) of RPDs. This corresponded to 3 (14.3%) of 21 complications of RPDs	Complications of artificial tooth: 5 (9.6%) of RPDs. This corresponded to 5 (23.8%) of complications of RPDs	Complications of denture base: 10 (19.2%) of RPDs. This corresponded to 10 (47.6%) of complications of RPDs
Mock, 2005 (parallel-sided double crowns with fricition fit) [23]	Objectifiable retention loss: 86% of satisfied patients; 39% of less satisfied patients (no figure can be given for the number because only the percentage is given and no reference value)	Decementation: 34 (37%) of patients			
Nickenig, 1995 (almost exclusively cylinder telescopic) [24]		Decementation: 39 (9.7%) of primary crowns, corresponding to 10.0 ± 3.3% of treatment measures at 5 years and 14.1 ± 5.1% at 8 years.		Damage to veneering: 25 (6.2%) of the primary crowns, corresponding to 4.7 ± 2.6% of post-treatment measures after 5 years and 7.1 ± 3.7% after 8 years.	

Rehmann, 2006 (cylinder telescopic RDPs, in a few cases (5.4%) combined with clasps on the molars) [27]	Improved friction: 41 (7.4%) of RPDs. This corresponded to 48 (2%) of all 2182 treatments	Recementation: 114 (21%) of RPDs. This corresponded to 216 (10%) of all treatment	Metal base repair: 14 (2.5%) of RPDs. This corresponded to 20 (1%) of all treatments. Primary crown or framework remake/repair: 39 (7%) of RPDs. This corresponded to 48 (2%) of all treatments	Facing repair: 149 (27%) of RPDs. This corresponded to 467 (22%) of all treatments. Resetting up a tooth: 63 (11.4%) of RPDs. This corresponded to 75 (3%) of all treatments. Reinserting a tooth: 61 (11%) of RPDs. This corresponded to 146 (7%) of all treatments	Acrylic base repair: 41 (7.4%) of RPDs. This corresponded to 54 (2%) of all treatments	Relining: 193 (35%) of RPDs. This corresponded to 347 (16%) of all treatments	Removing high spots: 210 (38%) of RPDs. This corresponded to 56 (26%) of all treatments
Schwindling, 2014 (telescopic, conical, resilient) [31]		Recementation: 40 (34.2%) of RPDs/47 (12.2%) of abutment teeth; 14 (35%) of RPDs with repeated recementation	Cracks or fractures in prothesis framework or base: 20 (17.1%) of RPDs	Reveneering: 13 (11.1%) of RPDs; 12 (92.3%) RPDs with multiple reveneering		Relining: 14 (12%) of RPDs	
Stober, 2020 (telescopic) [36]				Mean occlusal wear of RPD: 91 ± 85 μm (maximum: 329 ± 204 μm)			
Wagner, 2000 (conical crowns on anterior teeth and clasps on molars combined in a single denture) [41]	Retention: 1-10 N (mean: 5 N)						
Wagner, 2000 (all combined: clasp retained, conical crowns, conical crowns on anterior teeth			Fractures in metal: 8 (11.1%) of all RPDs	Facing lost: 16 (22.2%) of all RPDs. Facing discolored: 14 (19.4%) of all RPDs	Fractures in acrylic: 12 (16.7%) of all RPDs		

and clasps on molars combined in a single denture) [41]							
Wenz, 2001 (Marburger double crowns with TC-SNAP system) [42]			Fracture of metal framework: 0 (0%) of RPDs				
Widbom, 2004 (double-crown-retained with various numbers of replaceable snap attachments (Ipso-clips)) [43]		Abutment tooth fracture/loss of coping cement bond: 22 (6%) of the abutment teeth	Fracture of framework: 4 (5.3%) of RPDs				
Wöstmann, 2007 (cylinder telescopic RDPs, in a few cases (5.4%) combined with clasps on the molars) [45]	Increase in friction by apposition of alloy by laser welding into the secondary crown: 41 (7.4%) of RPDs. This corresponded to 48 (3%) of all 1626 treatments	Recementation: 114 (20.6%) of RPDs. This corresponded to 216 (13.3%) of all treatments	Repair of metal framework: 14 (2.5%) of RPDs. This corresponded to 20 (1.2%) of all treatments	Facing repair: 149 (26.9%) of RPDs. This corresponded to 467 (28.7%) of all treatments. Reattaching a denture tooth: 61 (11%) of RPDs. This corresponded to 146 (8.9%) of all treatments. Resetting up of denture teeth: 63 (11.4%) of RPD. This corresponded to 75 (4.6%) of all treatments	Acrylic base repair: 41 (7.4%) of RPDs. This corresponded to 54 (3.3%) of all treatments	Relining: 193 (34.8%) of RPDs. This corresponded to 347 (21.3%) of all treatments	Remake/repair of primary or secondary crowns: 39 (7%) of RPDs. This corresponded to 48 (3%) of all treatments
Yoshino, 2020 [46]				Need to remade because of prosthetic tooth wear: 2 (0.9%) of the RPDs	Need to remade because of incompatibility of mucosal surface: 10 (4,7%) of RPDs		

Zierden, 2018 (double-crowns) [47]	Retention reduction Initial: 95 (26%) of 600 cases in non-precious metal alloy (NPMA) RPDs; 15 (6.4%) cases in precious metal alloy (PMA) RPDs; follow-up: 112 (9.8%) cases in NPMA RPDs; 27 (2.1%) cases in PMA RPDs.<br>Increasing retention Initial: 6 (1.6%) cases in NPMA RPDs; 1 (0.4%) case in PMA RPDs; follow-up: 33 (2.9%) cases in NPMA RPDs; 68 (5.4%) cases in PMA RPDs	Recementation: initial maintenance intervention: 32 (8.7%) cases in non-precious metal alloy RPDs; 17 (7.3%) cases in precious metal alloy RPDs; maintenance intervention: 160 (14%) cases in non-precious metal alloy RPDs; 156 (12.3%) cases in precious metal alloy RPDs	Repair of metal framework: initial maintenance intervention: 0 (0%) case in non-precious metal alloy RPDs; 1 (0.4%) case in precious metal alloy RPDs; maintenance intervention: 57 (5%) cases in non-precious metal alloy RPDs; 45 (3.6%) cases in precious metal alloy RPDs. Renewal of metal framework: maintenance intervention: 16 (1.4%) cases for non-precious metal alloy RPDs; 26 (2.1%) cases for precious metal alloy RPDs.	New veneering of secondary crown: initial maintenance intervention: 8 (2.2%) cases in non-precious metal alloy RPDs; 17 (7.3%) cases in precious metal alloy RPDs; maintenance intervention: 102 (8.9%) cases in non-precious metal alloy RPDs; 138 (10.9%) cases in precious metal alloy RPDs	Adjustment/repair of acrylic material: initial maintenance intervention: 27 (7.4%) cases in non-precious metal alloy RPDs; 26 (11.1%) cases in precious metal alloy RPDs; maintenance intervention: 121 (10.6%) cases in non-precious metal alloy RPDs; 137 (10.8%) cases in precious metal alloy RPDs	Relining: initial maintenance intervention: 14 (3.8%) cases in non-precious metal alloy RPDs; 17 (7.3%) cases in precious metal alloy RPDs; maintenance intervention: 118 (10.4%) cases in non-precious metal alloy RPDs; 183 (14.4%) cases in precious metal alloy RPDs	Adjustment/ correction of occlusion Initial: 56 (15.3%) cases in NPMA RPDs; 52 (22.2%) cases in PMA RPDs; follow-up: 133 (11.6%) cases in NPMA RPDs; 188 (14.8%) cases in PMA RPDs. Manufacturing of new primary crowns Initial: 2 (0.6%) cases in NPMA alloy RPDs; 3 (1.3%) cases in PMA RPDs; follow-up: 9 (0.8%) cases in NPMA RPDs; 5 (0.4%) cases in PMA RPDs

Table S4. Summary of the results of the included studies: other RPDs (bars, etc.).

**Author, year (specifics anchoring form)**	**Retention of prothesis**	**Retention loss of anchor crowns (decementation)**	**Fracture of the framework (including the anchorage elements and their repair)**	**Fracture/repair of denture teeth or veneering**	**Fracture/repair of acrylic denture base/saddles**	**Relining/rebasing**	**Other failures/ complications**
Schulte, 1980 (Swinglock-RDP) [30]			Repair of framework: 4 (7.6%) of RPDs			Relining: 14 (26.4%) of RPDs	
Vermeulen, 1996 [40]			Survival related to fracture of prosthetic framework: extension base: upper after 5 years: 84 ± 9%; lower after 5 years: 80 ± 5%; lower after 10 years: 64 ± 8% tooth-supported base: upper after 5 years: 88 ± 8%; upper after 10 years: 59 ± 18%; lower after 5 years: 84 ± 5 %; lower after 10 years: 63 ± 10 %		Survival related to adjustment denture base: extension base: upper after 5 years: 72 ± 11%; upper after 10 years: 36 ± 17%; lower after 5 years: 29 ± 5%; lower after 10 years: 10 ± 5% tooth-supported base: upper after 5 years: 83 ± 9%; upper after 10 years: 66 ± 17%; lower after 5 years: 89 ± 4 %; lower after 10 years: 65 ± 10 %		
